# Supplementary material for: Male-killing symbiont damages host's dosage-compensated sex chromosome to induce embryonic apoptosis
Source: Nat Commun. 2016 Sep 21;7:12781. doi: 10.1038/ncomms12781 (PMC5036004; doi:10.1038/ncomms12781)
Supplement: Supplementary Information — Supplementary Figures 1-5, Supplementary Methods and Supplementary References [file ncomms12781-s1.pdf]

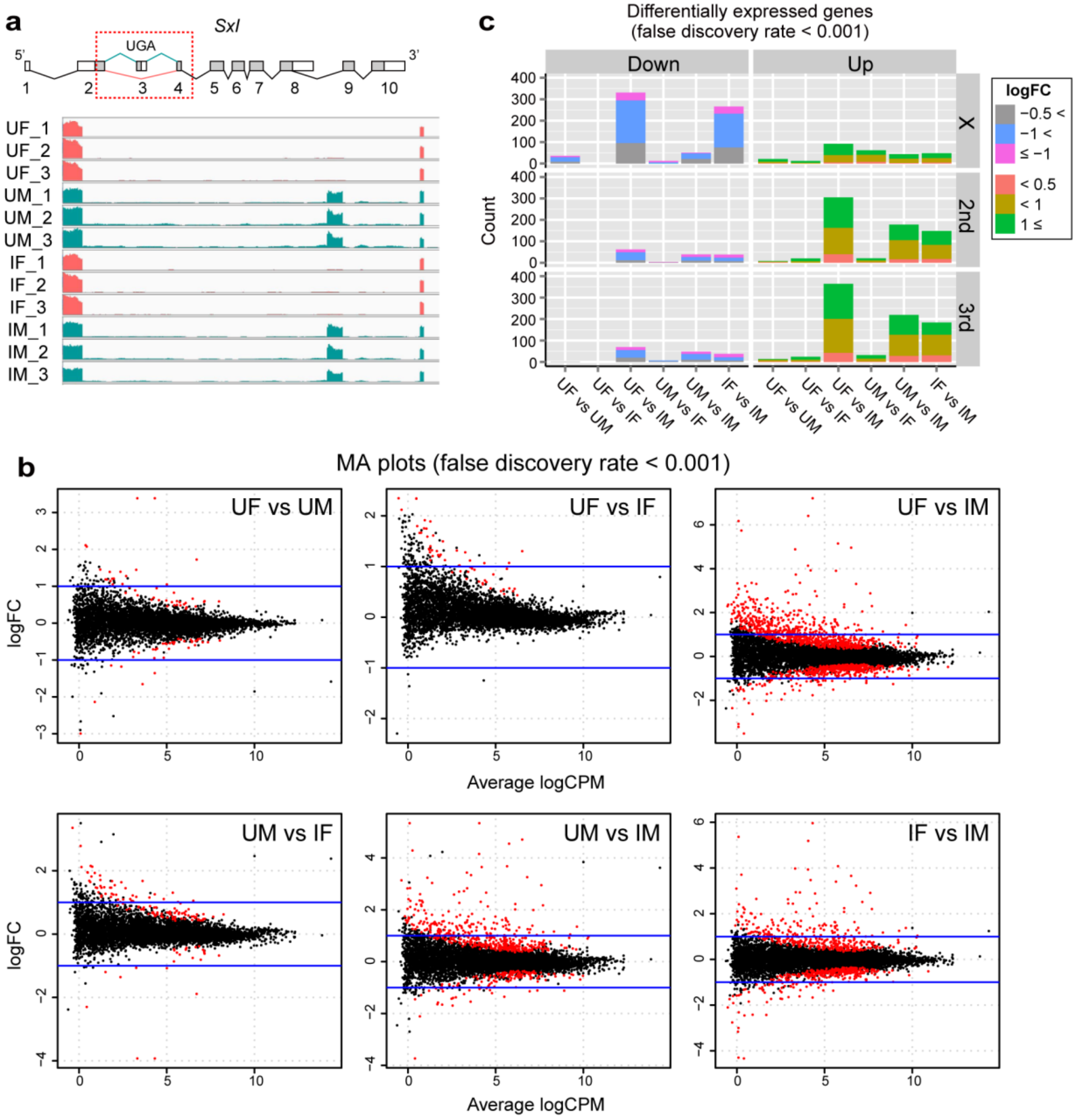

Supplementary Figure 1 | Differentially expressed genes among male and female embryos infected and uninfected with *Spiroplasma*.

(**a**) Results of short read mapping to the *Sxl* gene. The boxed region of the *Sxl* transcript (exon 2-4) is highlighted below. Only male samples (UM_1-3 and IM_1-3) exhibit mapped reads on the exon 3 with an internal UGA stop codon, which is skipped in females, confirming that embryonic sexing by *Sxl-Pe-EGFP* worked well. (**b**) MA plots between two samples. Log_2-transformed fold-changes (logFC) and averages of log_2-transformed counts per million reads (average logCPM) are plotted. Differentially expressed genes are highlighted in red and two-fold changes in expression levels between samples are shown in blue lines. (**c**) The number of differentially expressed genes plotted per chromosome. Genes down-regulated or up-regulated are separately plotted. Different colors represent the magnitude of the value of logFC. Genes on the Y and 4th chromosomes are omitted, because few genes are identified as differentially expressed on these chromosomes.

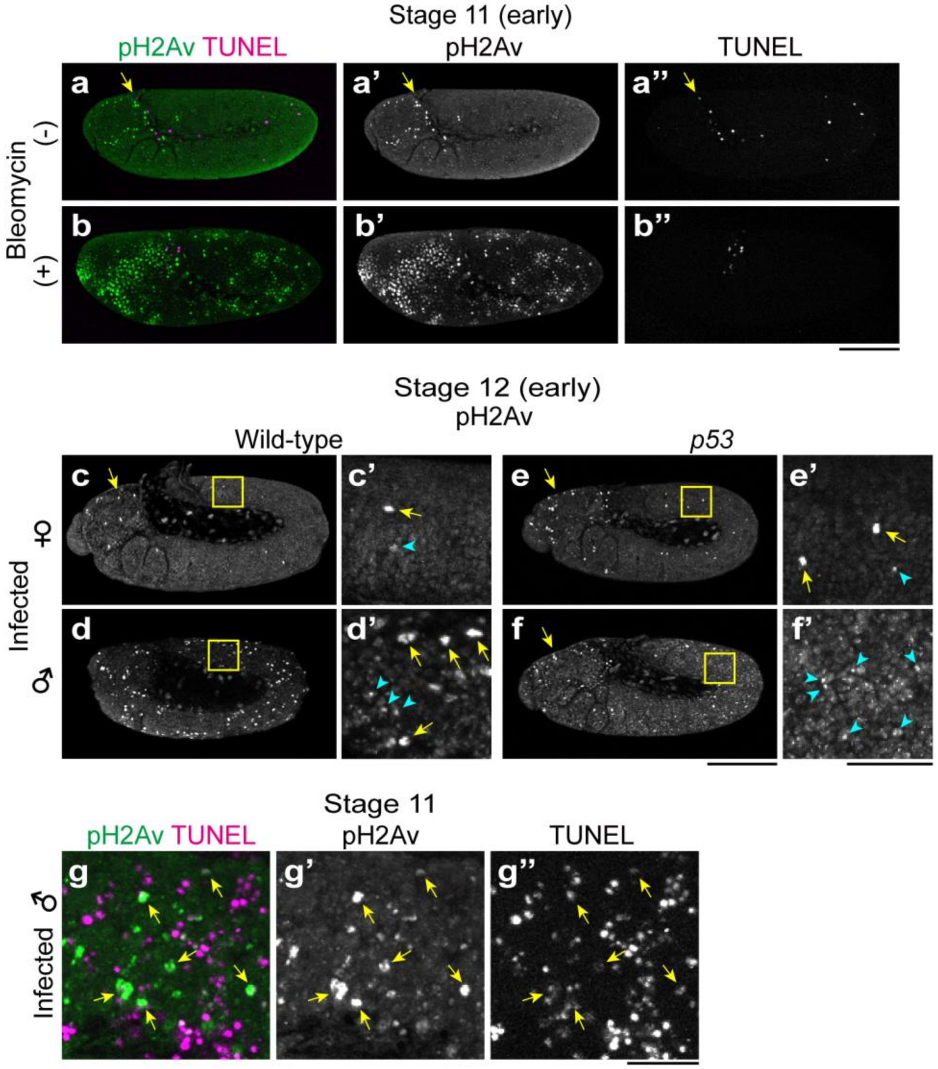

Supplementary Figure 2 | pH2Av antibody staining of *Drosophila* embryos.
(**a, b**) Early stage 11 embryos treated with or without a DNA damage-inducing drug bleomycin, which are stained for pH2Av (green) and TUNEL (magenta). Among bleomycin treated embryos (n = 143), 13% showed strong pH2Av signals throughout the body (**b-b''**). Probably, pH2Av-negative embryos were not sufficiently permeabilized and resulted in inefficient bleomycin incorporation. On the other hand, no ectopic pH2Av signals were observed in control embryos (**a-a''**, n = 165; arrows indicate pH2Av signals due to developmental apoptosis). In **b**, note that few pH2Av signals are overlapping with TUNEL signals, suggesting that these pH2Av signals represent DNA damage foci rather than abnormal apoptosis. (**c-f**) *Spiroplasma*-infected wild-type embryos (**c-c'**, female, n = 12; **d-d'**, male, n = 16) and *p53* mutant embryos (**e-e'**, female, n = 19; **f-f'**, male, n = 28) at early stage 12 stained for pH2Av. Boxed regions in **c-f** are magnified in **c'-f'**. Arrows in **c**, **e** and **f** denote developmental apoptosis in the head region. Yellow arrows and light blue arrowheads in **c'-f'** represent apoptotic signals and DNA damage foci, respectively. (**g**) High magnification image of epithelial cells of an infected male embryo stained for pH2Av (green) and TUNEL (magenta) (n = 16). Single channel images are shown in **g'** and **g''**. Arrows indicate overlaps between nuclear-wide pH2Av signals and TUNEL signals. Scale bars, 100 µm (**a-b'', c-f**) and 25 µm (**c'-f'**, **g-g''**).

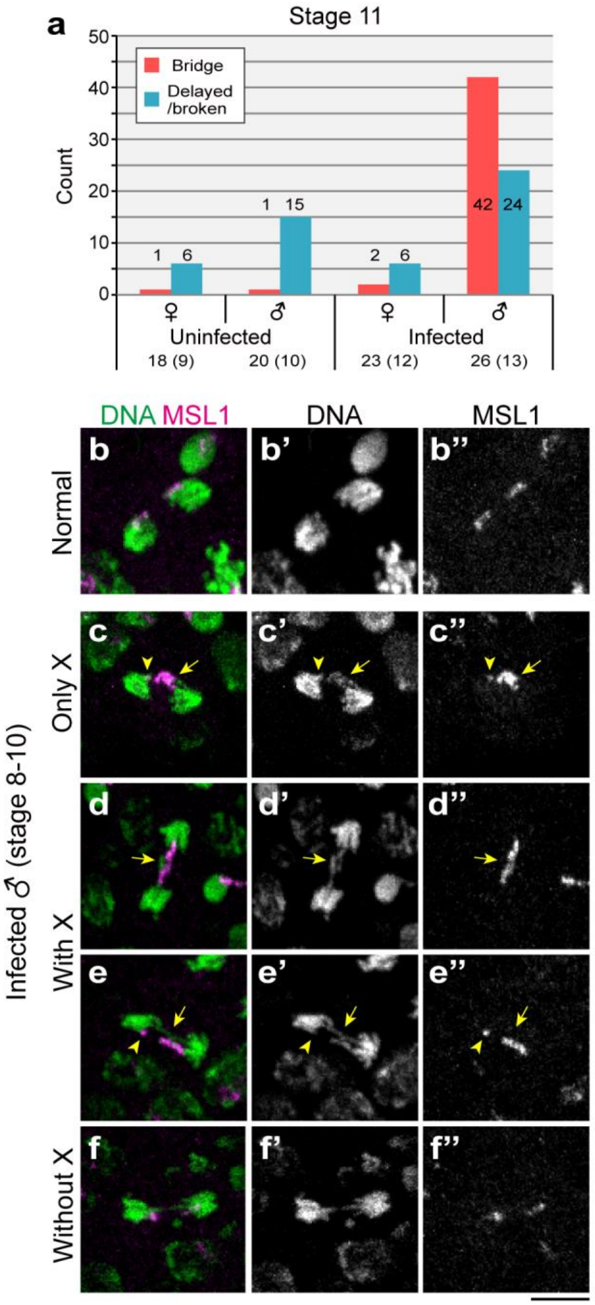

Supplementary Figure 3 | Examples of chromatin bridges observed in *Spiroplasma*-infected male embryos.

(**a**) The number of inter-nuclear bridges and delayed/broken chromosomes observed in *Spiroplasma*-infected and uninfected embryos at stage 11. The data set used for Fig. 3 and Fig. 4a-f was analyzed. Obtained images were categorized into two groups: (i) "bridge", which have two sister nuclei connected together with an obvious bridge (e.g. arrows in Fig. 4e, f), and (ii) "delayed/broken", which have slightly delayed chromosomes (e.g. arrows in Fig. 4c) or broken bridges that are apart from each other (we categorized these two phenotypes together, because we cannot distinguish them clearly). Sample sizes are shown at the bottom, and numbers of inspected embryos are shown in parentheses.

(**b-f)** Properly (**b**) and improperly (**c**-**f**) segregated sister chromatids from infected male embryos at stage 8-10 stained for DNA (green) and MSL1 (magenta) (n = 140 from 35 embryos). In most cases (83%; “only X”), bridges were entirely labeled with MSL1 (**c**; arrow and arrowhead indicating a tangled DNA mass and broken X chromatid, respectively). In some cases (15%; “with X”), bridges contained partially MSL-labeled chromosome (**d**; arrow) or consisted of multiple bridges with/without MSL1 staining (**e**; arrow and arrowhead indicating a bridge without MSL1 staining and broken X chromatid, respectively). On the other hand, bridges entirely lacking MSL1 signals were rare (**f**; 2%; “without X”). See the text for details. Scale bar, 5 μm.

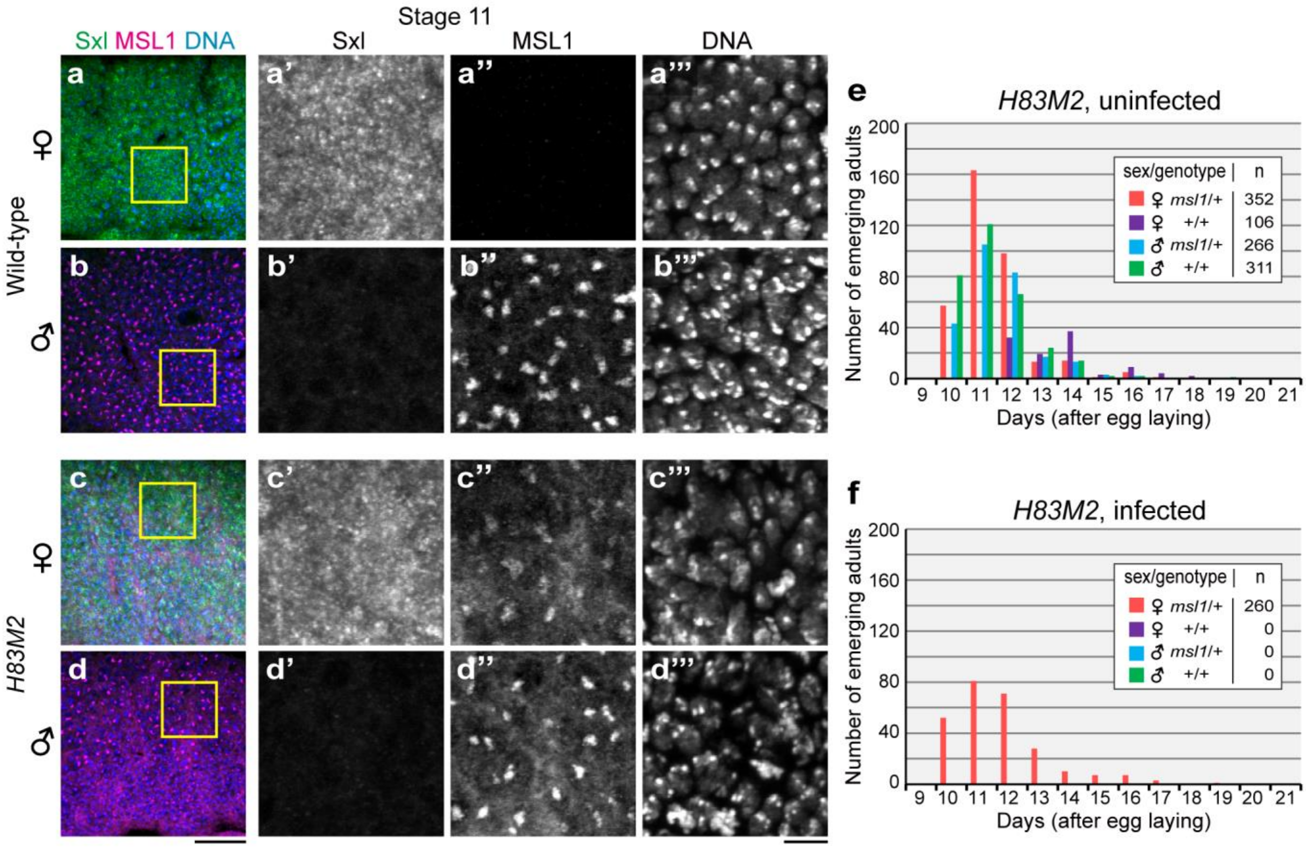

Supplementary Figure 4 | Ectopic MSL complex formation in *H83M2* female embryos.
(**a-d**) Wild-type embryos (**a-a'''**, female, n = 7; **b-b'''**, male, n = 7) and *H83M2* embryos (genotype +/*CyO ActGFP*; *H83M2*/+) (**c-c'''**, female, n = 7; **d-d'''**, male, n = 6) at stage 11, stained for Sxl (green), MSL1 (magenta) and DNA (blue). High magnification images of boxed regions in **a-d** are shown in **a'-d'''** as single channel images. (**e, f**) Adult emergence during 9-21 days after egg laying from crossing between females of wild-type genotype *Oregon-R* and males of genotype *msl1*^{L60}/*CyO ActGFP*; *H83M2* (**e**, uninfected; **f**, infected). Scale bars, 20 μm (**a-d**) and 5 μm (**a'-d'''**).

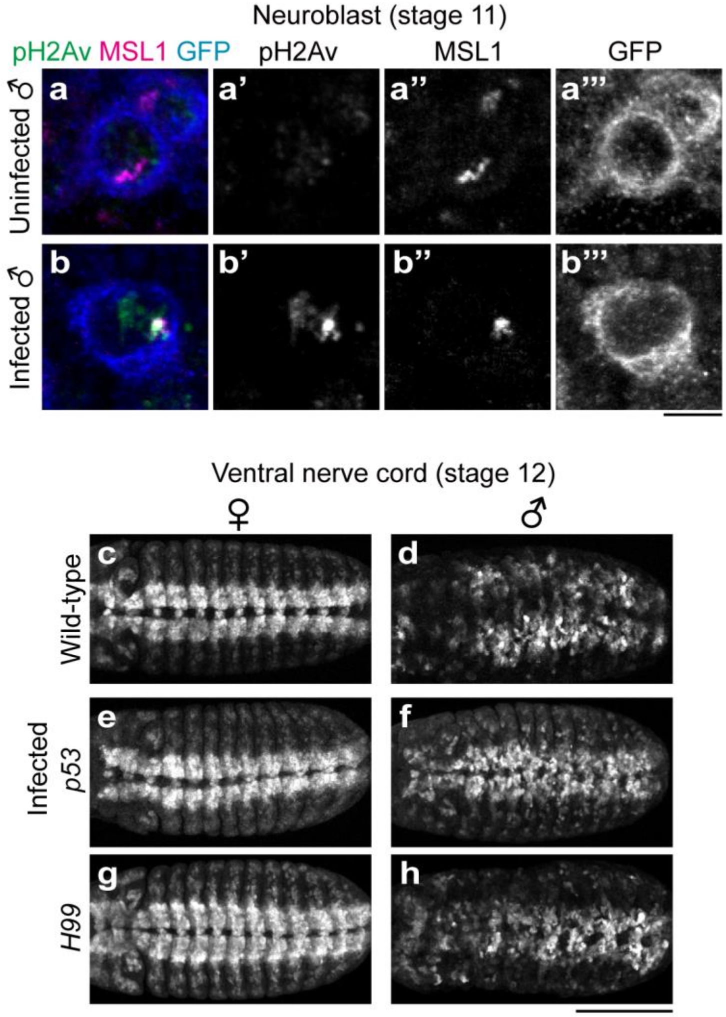

Supplementary Figure 5 | DNA damage in neural precursor cells and neural differentiation in *Spiroplasma*-infected *p53* mutant embryos.

(**a, b**) Uninfected (**a-a'''**, n =10) and infected (**b-b'''**, n = 9) male embryos at stage 11 stained for neuroblasts (blue; labeled by *31-1-GAL4 UAS-mCD8::GFP*), pH2Av (green) and MSL1 (magenta). (**c-h**) Infected wild-type embryos (**c**, female, n = 8; **d**, male, n = 8), *p53* mutant embryos (**e**, female, n = 16; **f**, male, n = 18), and apoptosis-deficient *H99* homozygous embryos (**g**, female, n = 8; **h**, male, n = 11) at stage 12 stained with anti-Elav antibody to detect differentiated neural cells in the ventral nerve cord. Scale bars, 5 µm (**a-b'''**) and 100 µm (**c-h**).

Supplementary Methods

Fly stocks and genetics

Laboratory strains of *D. melanogaster* were raised at 25°C on a standard cornmeal medium unless otherwise noted. *Oregon-R* (used as a wild-type strain) was provided by Takehide Murata (the Institute of Physical and Chemical Research, RIKEN). *Sxl-Pe-EGFP* G78b (ref. 1), *y w*; $p53^{5A\text{-}1\text{-}4}$ (ref. 2), and $msl3^{1}$ red^{1}/*TM3* Sb^{1} Ser^{1} (ref. 3) were obtained from the Bloomington *Drosophila* Stock Center (Indiana University). Strains carrying the green balancer (*CyO ActGFP* and *TM3 ActGFP* Ser^{1}), *Df(3L)H99* $kni^{ri\text{-}1}$ p^{p}/*TM3* Sb^{1} (ref. 4), *w*; *31-1-GAL4*/*TM6C* Sb^{1} Tb^{1} (ref. 5), and *y w*; Pin^{Yt}/*CyO*; *UAS-mCD8::GFP* (ref. 6) were obtained from the *Drosophila* Genetic Resource Center (DGRC) (Kyoto Institute of Technology). *p53R-GFPcyt* (ref. 7), $msl1^{L60}$/*CyO*; *H83M2* (ref. 8), and *stg* alleles (stg^{7B} and stg^{AR2})[9,10] were generously provided by John Abrams (University of Texas Southwestern Medical Center), Mitzi Kuroda (Harvard Medical School), and Bruce Edgar (Heidelberg University), respectively. To establish *Spiroplasma*-infected lines, fly strains were cultured in tetracycline containing medium (0.5-1.0 mg/ml) for one generation and infected with the male-killing *Spiroplasma* strain MSRO by hemolymph injection (see also ref. 11). MSRO-containing hemolymph was collected from a naturally *Spiroplasma*-infected *D. melanogaster* strain *Ug-SR* (ref. 12) provided by John Jaenike (University of Rochester).

$msl3^{1}$ and *H99* mutant strains were re-balanced with *TM3 ActGFP* Ser^{1} and segregated by staining with anti-GFP antibody to select homozygous or heterozygous embryos. To collect maternal mutant embryos of $msl3^{1}$, *Spiroplasma*-infected females of the genotype *w*; $msl3^{1}$ red^{1}/$msl3^{1}$ red^{1} were mated to males of the genotype *w*; $msl3^{1}$ red^{1}/*TM3 ActGFP* Ser^{1}. To produce female embryos expressing the ectopic MSL complex, *Spiroplasma*-infected and uninfected *Oregon-R* females were crossed to $msl1^{L60}$/*CyO ActGFP*; *H83M2* males. The resultant embryos were distinguished by GFP antibody staining, whereby only GFP-positive embryos (genotype +/*CyO ActGFP*; *H83M2*/+) were used except for the analysis in Supplementary Fig. 4e, f. In the experiments with *stg* mutants, *Spiroplasma*-infected stg^{AR2}/*TM3* females were crossed to stg^{7B}/*TM3* males, and resultant embryos were stained with anti-Discs large (Dlg) or anti-*D*α-catenin (DCAT-1) antibodies (see below) to check the size and number of cells (Fig. 6; see main text for details). To detect embryonic neuroblasts, *Spiroplasma*-infected and uninfected females of the genotype *w*; *31-1-GAL4 UAS-mCD8::GFP/TM3,* Sb^{1} were crossed to *Oregon-R* males and stained for GFP.

RNA extraction and RNA-seq library construction

Spiroplasma-infected and uninfected *Sxl-Pe-EGFP* embryos, collected at 5-7 h after egg laying were dechorionated and washed briefly by a washing buffer (0.7% NaCl, 0.1% Triton X-100). These embryos were observed under a stereo fluorescence microscope (Leica M165 FC) to determine the developmental stage 10-11 on the basis of morphology, and sexed on the criteria of GFP-positive females and GFP-negative males. Selected embryos were transferred to 1.5 ml tubes, from which washing buffer was removed completely, and were frozen in liquid nitrogen and stored at –80ºC. To minimize secondary effects of the experimental handling, embryo collection was completed within 30 min. Pooled embryos (around 90-230 per sample) were homogenized within RNAiso Plus reagent (TaKaRa) and purified by RNeasy Mini columns (QIAGEN). RNA-seq libraries were constructed by TruSeq RNA Sample Preparation Kit (Illumina). About 1 μg of total RNA was used for each library construction. RNA samples and constructed libraries were qualified by Agilent 2100 Bioanalyzer using RNA 6000 Nano Kit and High Sensitivity DNA Kit (Agilent Technologies), respectively. After quantification by Kapa Library Quantification Kit (Kapa Biosystems), all libraries were adjusted to a concentration around 10 nM and mixed for multiplex sequencing. Paired-end sequencing was performed using HiSeq 2000/2500 (Illumina) in the Dragon Genomics Center (TaKaRa) (for replicate #1) and in Functional Genomics

Facility in National Institute for Basic Biology (NIBB) (for replicates #2 and #3), respectively.

RNA-seq data analysis

Data analyses were performed using a 64-bit computer with Bio-Linux 7 operation software (NERC Environmental Bioinformatics Centre)[13]. The quality of raw sequence reads was checked by FastQC v0.11.4 (ref. 14). Short reads were aligned to the reference genome sequence of *D. melanogaster* provided by University of California, Santa Cruz (dm3, Berkeley *Drosophila* Genome Project Release 5) by TopHat v2.0.9 (ref. 15) with default options (Supplementary Data 2). Mapped reads were sorted by SAMtools v0.1.19 (ref. 16) and visualized in IGV v2.3.23 (ref. 17). Raw read counts were obtained by the HTSeq Python package v0.5.4 (ref. 18) with the intersection-nonempty mode. Differentially expressed genes were identified in all pairwise comparisons by using the edgeR Bioconductor package v3.4.2 with TMM (trimmed mean of M-values) normalization[19] (false discovery rate < 0.001). Of all genes annotated in the *Drosophila* genome, 8,387 genes achieved at least 1 CPM (counts per million reads) for at least 3 libraries were subjected to identification of differentially expressed genes (Supplementary Data 3). For hierarchical clustering, differentially expressed genes at relatively high expression levels (at least one sample with CPM $\geq 2^4$ and expression ratio ≥ 2; 320 of 1,430 differentially expressed genes) were clustered using the average linkage method using Cluster 3.0 (ref. 20). A heat map was generated by Java TreeView v1.1.6r4 with the helper script colorByThreshold.pl[21]. To obtain gene clusters, we cut the gene tree at the similarity of 0.65 and each cluster was subjected to GO analysis by the GOseq Bioconductor package v1.14.0 (ref. 22) (false discovery rate < 0.1). Finally, GO categories were summarized in the REVIGO website[23].

Immunofluorescent staining

Developmental staging of embryos was according to refs 24,25. Embryos were collected and stained as described previously[11]. In brief, embryos were collected from grape juice agar plates and dechorionated in 50% kitchen bleach, subsequently fixed in 1:1 mixture of heptane and 4% formaldehyde diluted in phosphate buffered saline [PBS; 137 mM NaCl, 2.7 mM KCl, 10 mM Na_2HPO_4 and 1.8 mM KH_2PO_4 (pH 7.4)] for 20 min, and devitellinized by vigorously shaking in heptane/MeOH. The embryos were washed in MeOH and rehydrated through an EtOH series, and then washed in PBT (PBS containing 0.1% Triton X-100). After treated with a blocking buffer [PBT containing 2% bovine serum albumin (BSA; Sigma-Aldrich, A7906)] for 30-60 min, the embryos were incubated with primary antibodies at 4ºC overnight, washed three times in PBT and incubated with secondary antibodies at room temperature for 90 min. Antibodies were diluted in the blocking buffer. Anti-Sex-lethal and anti-MSL1 antibodies were utilized for sexing embryos (see below). The following primary antibodies were used: mouse anti-Sex-lethal [1:20; Developmental Studies Hybridoma Bank (DSHB), M18][26], rabbit anti-Histone H2AvD pS137 (1:300; Rockland Immunochemicals Inc., 600-401-914), mouse and rabbit anti-MSL1 [1:200; kindly provided by John Lucchesi (Emory University)], rat anti-Elav (1:20; DSHB, 7E8A10)[27], chicken anti-GFP (1:400; Aves Labs, Inc., GFP-1020), mouse anti-Discs large (1:20; DSHB, 4F3)[28], and rat anti-*D*α–catenin (1:10; DSHB, DCAT-1)[29]. Secondary antibodies were purchased from Jackson ImmunoResearch Laboratories, Inc. and Molecular Probes. DAPI (4',6-diamidino-2-phenylindole) and SYTOX Orange Nucleic Acid Stain (1:20,000; Molecular Probes, S-11368) were used for DNA staining. TUNEL staining was performed as described[30] by using the In Situ Cell Death Detection Kit, TMR red (Roche Applied Science), and the embryos stained with primary antibodies were incubated in 50 µl TUNEL reaction mixture with secondary antibodies at 4ºC overnight. Stained embryos were washed three times in PBT, mounted in FluorSave Reagent (Calbiochem) and observed under a confocal microscope (Zeiss LSM 5 Pascal or LSM 510 META).

Bleomycin treatment of embryos

Bleomycin treatment was performed as described[31] with minor modification. Briefly, *Oregon-R* embryos (4-6 h after egg laying) were dechorionated and washed by the washing buffer. These embryos were treated in a 1:1 mixture of heptane and PBS with 100 μg/ml bleomycin (LKT Laboratories Inc., 9041-93-4) for 30 min at room temperature with vigorous shaking (300 rpm). Subsequently, PBS was replaced by 4% formaldehyde for fixation. The fixed embryos were devitellinized and stained by our standard staining protocol (see above).

Imaging analysis

Confocal z-sections were max projected by ImageJ software (National Institutes of Health). TUNEL signals and focal pH2Av signals were quantitatively analyzed by custom R scripts with the EBImage package for image processing[32]. TUNEL signals of whole embryos (acquired by a 20x/0.5 objective) were quantified and analyzed as described[11]. In brief, maximum projection images of Sxl staining were binarized to make mask images of embryos. Projected TUNEL images were also binarized and signals inside corresponding mask images were counted by image integration. These values were divided by mask image area for normalization. For quantitative analysis of focal pH2Av signals, images with focal pH2Av signals and MSL1 signals were acquired by a 63x/1.4 oil immersion objective with 2x zoom scan. Typically, two images were acquired per embryo. For making projected images of pH2Av and MSL1 signals, we collected and compiled 15 serial z-sections (with 0.4 μm intervals). These images were smoothed by Gaussian filter and binarized by moving average method, respectively. To identify focal pH2Av signals, image objects were extracted from the pH2Av images by segmentation and labeling, where objects larger than 100 pixels were eliminated to exclude apoptosis-derived signals (e.g. arrowheads in Fig. 3d). To calculate the enrichment of focal pH2Av signals on the male X chromosome, overlaps between focal pH2Av signals and MSL1 signals were obtained by image integration. Pairwise statistical comparisons were performed using the Mann-Whitney U test or Pearson's χ^2 test with Yates' continuity correction. Multiple comparisons were performed using the Kruskal-Wallis test followed by Mann-Whitney U tests with Holm adjustment. We used the R software v3.0.2 (ref. 33) for all statistical analyses.

Abnormal chromosomes during cell division were counted manually. For analysis of *Spiroplasma*-infected and uninfected *Oregon-R* embryos (Fig. 4i and Supplementary Fig. 3a), images were taken by a 63x/1.4 oil immersion objective with 2x zoom scan. Several images (1-5 depending on the embryo) were taken per embryo. In Fig. 4i, only anaphase bridges were counted and categorized into three groups as described in the main text. In Supplementary Fig. 3a, inter-nuclear bridges and delayed/broken chromosomes during anaphase and telophase were analyzed. For analysis of *H83M2* female embryos (Fig. 5k), images were taken by a 63x/1.4 oil immersion objective without zoom scan and inter-nuclear bridges during anaphase and telophase were counted.

Supplementary References

1. Thompson, J., Graham, P., Schedl, P. & Pulak, R. Sex-specific GFP-expression in *Drosophila* embryos and sorting by COPAS flow cytometry technique. *45th ADRC* (2004).

2. Rong, Y. S. *et al.* Targeted mutagenesis by homologous recombination in *D. melanogaster*. *Genes Dev.* **16,** 1568–1581 (2002).

3. Uchida, S., Uenoyama, T. & Oishi, K. Studies on the sex-specific lethals of *Drosophila melanogaster*. III. A third chromosome male-specific lethal mutant. *Jpn. J. Genet.* **56,** 523–527 (1981).

4. White, K. *et al.* Genetic control of programmed cell death in *Drosophila*. *Science* **264,** 677–683 (1994).

5. Brand, A. H. & Perrimon, N. Targeted gene expression as a means of altering cell fates and generating dominant phenotypes. *Development* **118,** 401–415 (1993).

6. Lee, T. & Luo, L. Mosaic analysis with a repressible cell marker for studies of gene function in neuronal morphogenesis. *Neuron* **22,** 451–461 (1999).

7. Lu, W.-J., Chapo, J., Roig, I. & Abrams, J. M. Meiotic recombination provokes functional activation of the p53 regulatory network. *Science* **328,** 1278–1281 (2010).

8. Kelley, R. L. *et al.* Expression of Msl-2 causes assembly of dosage compensation regulators on the X chromosomes and female lethality in *Drosophila*. *Cell* **81,** 867–877 (1995).

9. Edgar, B. A. & O'Farrell, P. H. Genetic control of cell division patterns in the *Drosophila* embryo. *Cell* **57,** 177–187 (1989).

10. Edgar, B. A., Lehman, D. A. & O'Farrell, P. H. Transcriptional regulation of *string* (*cdc25*): a link between developmental programming and the cell cycle. *Development* **120,** 3131–3143 (1994).

11. Harumoto, T., Anbutsu, H. & Fukatsu, T. Male-killing *Spiroplasma* induces sex-specific cell death via host apoptotic pathway. *PLoS Pathog.* **10,** e1003956 (2014).

12. Pool, J. E., Wong, A. & Aquadro, C. F. Finding of male-killing *Spiroplasma* infecting *Drosophila melanogaster* in Africa implies transatlantic migration of this endosymbiont. *Heredity (Edinb)* **97,** 27–32 (2006).

13. Field, D. *et al.* Open software for biologists: from famine to feast. *Nat. Biotechnol.* **24,** 801–803 (2006).

14. Andrews, S. FastQC: a quality control tool for high throughput sequence data. (2010). Available at: http://www.bioinformatics.babraham.ac.uk/projects/fastqc/.

15. Trapnell, C. *et al.* Differential gene and transcript expression analysis of RNA-seq experiments with TopHat and Cufflinks. *Nat. Protoc.* **7,** 562–578 (2012).

16. Li, H. *et al.* The Sequence Alignment/Map format and SAMtools. *Bioinformatics* **25,** 2078–2079 (2009).

17. Thorvaldsdóttir, H., Robinson, J. T. & Mesirov, J. P. Integrative Genomics Viewer (IGV): high-performance genomics data visualization and exploration. *Brief. Bioinformatics* **14,** 178–192 (2013).

18. Anders, S., Pyl, P. T. & Huber, W. HTSeq--a Python framework to work with high-throughput sequencing data. *Bioinformatics* **31,** 166–169 (2015).

19. Robinson, M. D., McCarthy, D. J. & Smyth, G. K. edgeR: a Bioconductor package for differential expression analysis of digital gene expression data. *Bioinformatics* **26,** 139–140 (2010).

20. de Hoon, M. J. L., Imoto, S., Nolan, J. & Miyano, S. Open source clustering software. *Bioinformatics* **20,** 1453–1454 (2004).

21. Saldanha, A. J. Java Treeview--extensible visualization of microarray data. *Bioinformatics* **20,** 3246–3248 (2004).

22. Young, M. D., Wakefield, M. J., Smyth, G. K. & Oshlack, A. Gene ontology analysis for RNA-seq: accounting for selection bias. *Genome Biol.* **11,** R14 (2010).

23. Supek, F., Bošnjak, M., Škunca, N. & Šmuc, T. REVIGO summarizes and visualizes long lists of gene ontology terms. *PLoS ONE* **6,** e21800 (2011).

24. Campos-Ortega, J. A. & Hartenstein, V. *The Embryonic Development of Drosophila melanogaster*. (Springer, 1985).

25. Hartenstein, V. *Atlas of Drosophila Development*. (Cold Spring Harbor Laboratory Press, 1993).

26. Bopp, D., Bell, L. R., Cline, T. W. & Schedl, P. Developmental distribution of female-specific Sex-lethal proteins in *Drosophila melanogaster*. *Genes Dev.* **5,** 403–415 (1991).

27. O'Neill, E. M., Rebay, I., Tjian, R. & Rubin, G. M. The activities of two Ets-related transcription factors required for *Drosophila* eye development are modulated by the Ras/MAPK pathway. *Cell* **78,** 137–147 (1994).

28. Parnas, D., Haghighi, A. P., Fetter, R. D., Kim, S. W. & Goodman, C. S. Regulation of postsynaptic structure and protein localization by the Rho-type guanine nucleotide exchange factor dPix. *Neuron* **32,** 415–424 (2001).

29. Oda, H. *et al.* Identification of a *Drosophila* homologue of alpha-catenin and its association with the armadillo protein. *J. Cell Biol* **121,** 1133–1140 (1993).

30. Krieser, R. J. *et al.* The *Drosophila* homolog of the putative phosphatidylserine receptor functions to inhibit apoptosis. *Development* **134,** 2407–2414 (2007).

31. Takada, S., Kwak, S., Koppetsch, B. S. & Theurkauf, W. E. *grp* (*chk1*) replication-checkpoint

mutations and DNA damage trigger a Chk2-dependent block at the *Drosophila* midblastula transition. *Development* **134,** 1737–1744 (2007).

32. Pau, G., Fuchs, F., Sklyar, O., Boutros, M. & Huber, W. EBImage--an R package for image processing with applications to cellular phenotypes. *Bioinformatics* **26,** 979–981 (2010).

33. R Core Team. R: a language and environment for statistical computing. R Foundation for Statistical Computing, Vienna, Austria. URL https://www.R-project.org/. (2013).
